# lncRNA SNHG15 as a ceRNA modulates Osteoclast Differentiation, Proliferation, and Metastasis by Sponging miR-381-3p/NEK2 Axis

**DOI:** 10.1155/2022/8634820

**Published:** 2022-06-12

**Authors:** YiFan Wang, GuanYin Zhu, Fang Pei, ZhiHe Zhao

**Affiliations:** ^1^State Key Laboratory of Oral Diseases & National Clinical Research Center for Oral Diseases, West China Hospital of Stomatology, Sichuan University, No. 14, 3rd Section, South Renmin Road, Chengdu, Sichuan 610041, China; ^2^Department of Orthodontics, West China Hospital of Stomatology, Sichuan University, Chengdu, Sichuan, China

## Abstract

**Background:**

A growing number of studies have shown that long noncoding RNAs play an important role in osteoclast differentiation. However, there are few studies on the roles of lncRNA small nucleolar RNA host gene 15 (SNHG15) in osteoclast differentiation.

**Methods:**

The expressions of SNHG15, miR-381-3p, and never in mitosis-related kinase 2 (NEK2) mRNA were detected by real-time quantitative polymerase chain reaction (RT-qPCR); Western blot detected NEK2 and osteoclast markers (Cathepsin K, CTSK), matrix metalloproteinase 9 (MMP9), nuclear factor of activated T cell 2 (NFAT2), and tartrate-resistant acid phosphatase (TRAP) protein levels; cell proliferation was detected by Cell Counting Kit-8 (CCK-8), and the formation of osteoclasts was observed by TRAP staining; the F-actin skeleton was stained with tetramethylrhodamine isothiocyanate (TRITC) phalloidin; cell migration rate was detected by Transwell; dual-luciferase reporter gene assay and RNA-binding protein immunoprecipitation (RIP) assay verified the targeting relationship between miR-381-3p, SNHG15, and NEK2.

**Results:**

The expression of SNHG15 was increased in THP-1 cells stimulated by macrophage colony-stimulating factor (M-CSF)/receptor activator of nuclear factor-kappa B ligand (RANKL). Overexpression of SNHG15 significantly promoted the proliferation, migration, osteoclast differentiation, and expression of osteoclast markers CTSK, MMP9, NFAT2, and TRAP of THP-1 cells induced by M-CSF/RANKL. Knockdown of SNHG15 reversed this effect. Overexpression of SNHG15 downregulated the inhibitory effect of overexpression of miR-381-3p on the proliferation, migration, and differentiation of THP-1 cells induced by M-CSF/RANKL. Knockdown of miR-381-3p reversed the inhibitory effect of knockdown of NEK2 on the proliferation, migration, and differentiation of THP-1 cells induced by M-CSF/RANKL.

**Conclusion:**

SNHG15 acted as a ceRNA promoted the proliferation, migration, and differentiation of THP-1 cells induced by M-CSF/RANKL through sponging miR-381-3p to promote the expression of NEK2.

## 1. Introduction

Osteoclasts are fusion-differentiated mononuclear macrophages from hematopoietic stem cells that produce tartrate-resistant acid phosphatase (TRAP) [1]. Osteoclasts are the only cells with the ability to degrade tissues, regulating bone resorption. Osteoblasts are the multinucleated cells derived from the monocyte/macrophage lineage [2] and are mainly responsible for regulating bone formation, while the synthesis of cytokines such as macrophage colony stimulating factor (M-CSF), monocyte chemoattractant protein-1 (MCP-1), and receptor activator of nuclear factor-kappa B ligand (RANKL) regulates osteoclast formation [3]. Under normal circumstances, osteoblasts and osteoclasts are in dynamic balance to jointly maintain the stability of bone mechanics. Once this balance is disrupted, it will lead to a series of bone tissue diseases including osteosclerosis and osteoporosis. Recent studies have reported that noncoding RNAs are involved in the regulation of osteoclast differentiation [4]. However, the specific regulatory mechanism remains to be further studied.

Long noncoding RNAs (lncRNAs) are noncoding RNAs that are more than 200 nucleotides in length and do not have protein-coding capabilities. lncRNAs are involved in regulating gene transcription, mRNA splicing, and histone modification through various ways such as cis-regulation and transregulation to promote the activation of transcription factors and inhibit the expression of downstream genes. Among them, as competing endogenous RNA (ceRNA), lncRNAs compete with miRNAs through miRNA response elements (MREs) to inhibit the expression and activity of miRNAs, thereby inhibiting the degradation of targeting mRNA, which is the main regulation mechanism of lncRNA. There are many researches of the late that have reported that lncRNAs are abnormal expression in osteoclast. For example, Shao et al. [5] reported that knockdown of XIST inhibited the differentiation of osteoclast; the expression of lncRNA AK077216 is elevated in the genesis of osteoclast [6]. lncRNA small nucleolar RNA host gene 15 (SNHG15) is the lncRNA located on human chromosome 7 and has been demonstrated upregulation in a variety of tumors, such as breast cancer [7], hepatocellular carcinoma [8], and nasopharyngeal carcinoma [9]. Liu and Huang [10] reported that SNHG15 participated in the regulation of osteoclast. However, the function of SNHG15 in osteoclast is unclear.

miRNAs are shot endogenous noncoding RNAs and regulate the gene expression of posttranscriptional through binding with the 3′-untranslated regions (3′-UTR) of target mRNA. A large deal of reports have certificated miRNAs participate in the regulation of cell biological behaviors such as differentiation [11], proliferation [12], metastasis [13], and apoptosis [14]. Among osteoclast-associated miRNAs, miR-214-3p has been confirmed to be highly expressed in osteoclasts and inhibited the osteoblast activity *in vitro* [15]; the expression of miR-195a was decreased in osteoclast and reversed the effect of circRNA-28313 on osteoclast differentiation [16]. However, the role of miR-381-3p in osteoclast is obscure.

In this study, we explored the specific regulatory mechanism of lnc SNHG15 on osteoclast proliferation, differentiation, and metastasis and experimentally confirmed the increased expression of SNHG15 in osteoclasts. Overexpression of SNHG15 promoted the proliferation, differentiation, and metastasis of osteoclasts through sponging with miR-381-3p to upregulate the expression of NEK2.

## 2. Materials and Methods

### 2.1. Cell Lines and Cell Culture

THP-1 cells (Procell, China) were cultured in RPMI-1640 medium (Procell, China) containing 10% fetal bovine serum (FBS), 0.05 mM *β*-mercaptoethanol and 1% PBS. 293T cells (Procell, China) were cultured in DMEM medium (Procell, China) containing 10% FBS and 1% penicillin/streptomycin. Then, the culture medium was placed in a 5% CO_2_ cell incubator at 37°C.

### 2.2. Induced of Osteoclast

THP-1 cells were seed into 6-well plates. 200 ng/mL phorbol-12-myristate-13-acetate (PMA, Sigma-Aldrich, MO, USA) was used to induce THP-1 cell differentiation into macrophages. 25 ng/mL M-CSF (PeproTech, NJ, USA) and 30 ng/mL RANKL (PeproTech, NJ,USA) were added into the plates to induce osteoclast-like cells, and the cell differentiation was detected by experiment 14 days later [17].

### 2.3. Cell Transfection

Sh-NC, sh-SNHG15, pcDNA-NC, pcDNA-SNHG15, NC-mimics, miR-381-3p inhibitor, NC-inhibitor, miR-381-3p inhibitor, miR-381-3p mimic+pcDNA-SNHG15, sh-NEK2, and sh-NEK2+miR-381-3p inhibitor were transfected into RANKL/M-CSF-stimulated THP-1 cells, respectively. The cells were cultured in a cell incubator for 48 h.

### 2.4. Real-Time Quantitative Polymerase Chain Reaction (RT-qPCR)

The cells of each group were collected; TRIzol reagent (Thermo Fisher, MA, USA) was added to lysis the cells and extracted total RNA. Ultraviolet spectroscope (Beckman, FL, USA) detected the concentration and purity of RNA. According to the instruction book (Thermo Fisher, MA, USA), RT-qPCR was performed. The reaction condition was 95°C predenaturation for 3 min, 95°C denaturation for 30 s, 72°C anneal for 60 s, a total of 34 cycles, and 72°C full extensions 10 min. GAPDH served as the internal reference of SNHG15; U6 served as the internal reference of miR-381-3p. 2^−ΔΔCt^ methods analysed the data. The primer sequences were as follows (Table 1).

### 2.5. Western Blot

The total protein was extracted from cells of each group by RIPA reagent (Beyotime, China), respectively. The methods of Bradford detected the content of protein. Protein was separated by 12% sodium dodecyl sulfate polyacrylamide gel electrophoresis and then transferred to PVDF membrane (Beyotime, China). 5% skimmed milk powder (Solaibio, China) was added to incubate for 1 h at 37°C. Primary antibody (Abcam, UK; Cathepsin K (CTSK): 1 *μ*g/mL; matrix metalloproteinase 9 (MMP9): 1 : 1000; nuclear factor of activated T cell 2 (NFAT2): 1 *μ*g/mL; TRAP: 1 : 5000) was added and incubated overnight at 4°C. Then, second antibody (Abcam, UK) was added and incubated at room temperature for 2 h. After rinsing the membrane, developing liquid was added for color development and fixing. Image J analysed the gray value of the strip.

### 2.6. Cell Counting Kit-8 (CCK-8)

The suspended cells were inoculated into 96-well (Corning, NY, USA) plate at a density of 5 × 10^3^ cells/well and cultured in a cell incubator for an appropriate time (48 h). At the corresponding time point, 10 *μ*L CCK-8 reagent (Solaibio, China) was added to each well. The absorbance value at 450 nm was detected by a microplate reader (Thermo Fisher, MA, USA) after reaction for 2 h.

### 2.7. Transwell Assay

The culture medium of RPMI-1640 (600 *μ*L) was added into each well of the 24-well (Corning, NY, USA) plate for culture, and the attached-wall cells were digested by trypsin. Then, RPMI-1640 was added in to adjust the cell concentration to 1 × 10^4^/mL suspension, and 200 *μ*L of the prepared cell suspension was inoculated into a Transwell (Corning, NY, USA) upper chamber for spreading and immersed in a 24-well plate medium for culture for 24 h. After that, the cells were rinsed with PBS and fixed with methanol for 10 min. The cotton swabs were used to wipe off the cells attached to the membrane in the upper chamber of Transwell, and the sections were packaged and observed under the microscope.

### 2.8. RNA-Binding Protein Immunoprecipitation (RIP) Assay

The collected cells were washed by PBS and centrifuged by Eppendorf tube (Hamburg, Germany), the supernatant was discarded, and the cells were resuspended by RIP lysate (Millipore, MA, USA). The RIP wash buffer magnetic beads were resuspended, and the corresponding antibodies were added and incubated for 30 min at room temperature. The cell lysis supernatant, magnetic bead-antibody complex, and RNA immunoprecipitation buffer were mixed and incubated overnight at 4°C. After the RNA was rinsed by RIP wash buffer, phenol, chloroform, and isopropyl alcohol were added for purification, and 80% ethanol was used to wash for RT-qPCR detection.

### 2.9. Dual-Luciferase Reporter Gene Assay

Wild type pGL3-WT and mutant type pGL3-MUT plasmid (GenePharma, China) of SNHG15 and NEK2 was constructed, respectively. StarBase database predicted the target binding sequence of SNHG15-miR-381-3pandmiR-381-3p-NEK2. To further verify the target-binding relationship between them, miR-381-3p mimics, NC mimics, SNHG15-WT/MUT, and NEK2-WT/MUT were transfected into 293T cell according to the conduction of luciferase assay kit (Promega, WI, USA), respectively. Dual-luciferase report gene methods detected the luciferase activity of each group.

### 2.10. Statistical Analysis

GraphPad Prism 8.0 was used to analyzed the data and graph. All data were expressed as mean ± standard deviation, Student's *T* test was used for comparison between two groups, and one-way ANOVA was used for comparison between multiple groups. *P* < 0.05 represented the difference was statistically significant.

## 3. Results

### 3.1. Induction of Osteoclasts and the Expression of lnc SNHG15 and miR-381-3p in Osteoclasts

**Figure 1:**
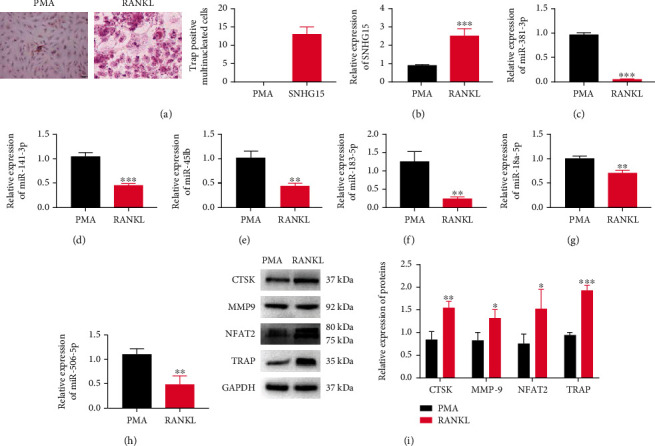
Induction of osteoclasts and the expression of lnc SNHG15 and miR-381-3p in osteoclasts. (a) TRAP staining observed osteoclast of THP-1 cells induced by RANKL; (b–h) RT-qPCR detected the expression of SNHG15, miR-381-3p, miR-141-3p, miR-451b, miR-183-5p, miR-18a-5p, and miR-506-5p; (i) Western blot detected the proteins expression of osteoclast markers CTSK, MMP-9, NFAT2, and TRAP. Compared with PMA group: ^∗^*P* < 0.05, ^∗∗^*P* < 0.01, and ^∗∗∗^*P* < 0.001.

THP-1 cells were induced to differentiate into macrophages by PMA (PMA group) and then induced macrophages into osteoclasts by M-CSF and RANKL (RANKL group). We stained the induced cells with TRAP and found that compared to the PMA group, TRAP(+) multinuclear cells in the RANKL group were increased (Figure 1(a)), indicating the successful induction of osteoclasts. Subsequently, we detected the relative mRNA expressions of lncSNHG15 and its potential target genesmiR-381-3p, miR-141-3p, miR-451b, miR-183-5p, miR-18a-5p, and miR-506-5p by RT-qPCR. Compared with the Ctrl group, SNHG15 was highly expressed in the RANKL group (Figure 1(b)), while miR-381-3p (Figure 1(c)), miR-141-3p (Figure 1(d)), miR-451b (Figure 1(e)), miR-183-5p (Figure 1(f)), miR-18a-5p (Figure 1(g)), and miR-506-5p (Figure 1) were decreased in the RANKL group, and miR-381-3p was significantly lower than other miRNAs. Western blot analysis showed that the levels of osteoclast markers CTSK, MMP-9, NFAT2, and TRAP in the RANKL group were significantly higher than those in the PMA group. In summary, we successfully induced osteoclasts. At the same time, we found that SNHG15 and miR-381-3p were abnormally expressed in osteoclasts, and they might be involved in the regulation of osteoclast differentiation.

### 3.2. Effects of SNHG15 on Osteoclast Differentiation, Proliferation, and Movement

**Figure 2:**
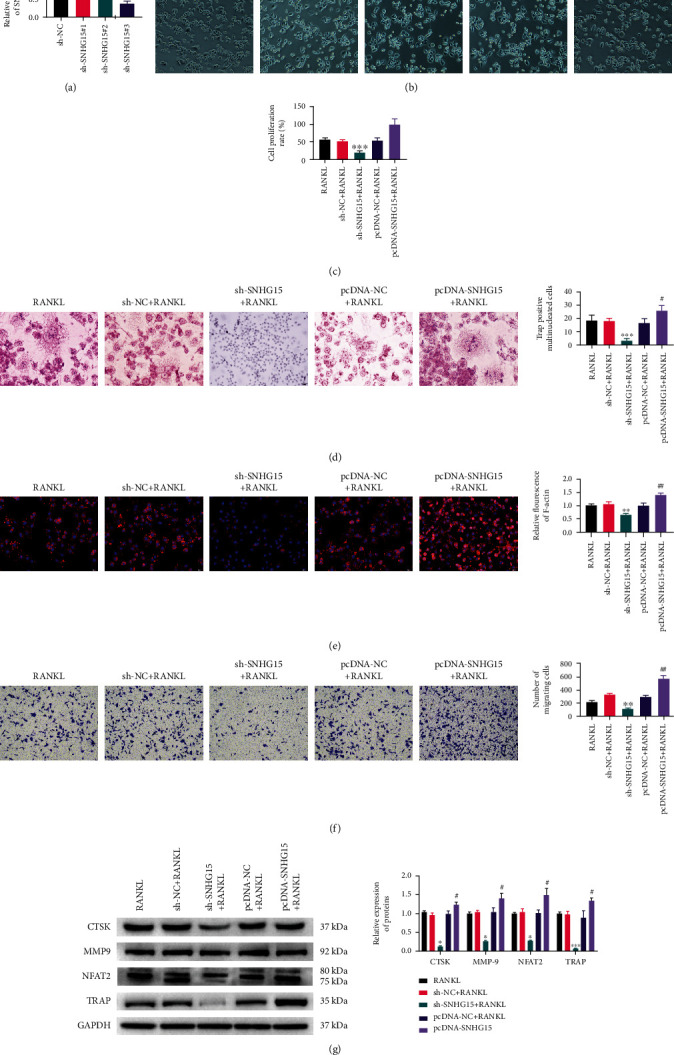
Effects of SNHG15 on osteoclast differentiation, proliferation, and movement of osteoclast precursors. (a) The transfection of sh-SNHG15 in 293T cells was detected by RT-qPCR; (b) cell morphology was observed by optical microscope; (c) CCK-8 was used to detect cell proliferation rate; (d) TRAP staining observed osteoclast of THP-1 cells; (e) rhodamine-labeled phalloidin staining observed the positive rate of F-actin. (f) Transwell detected cell migration; (g) Western blot was used to detect proteins expression of osteoclast markers CTSK, MMP-9, NFAT2, and TRAP. Compared with sh-NC group: ^&^*P* < 0.05, ^&&&^*P* < 0.001; compared with RANKL+sh-NC group: ^∗^*P* < 0.05, ^∗∗^*P* < 0.01, and ^∗∗∗^*P* < 0.001; compared with RANKL+pcDNA-NC group: ^#^*P* < 0.05, ^##^*P* < 0.01.

We transfected 293T cells with sh-NC, sh-SNHG15#1, sh-SNHG15#2, and sh-SNHG15#3, respectively. RT-qPCR assay showed that SNHG15 expression was decreased in the transfected sh-SNHG15 group (Figure 2(a)), and SNHG15 expression was lower in the sh-SNHG15#3 group than in the other transfected groups. Therefore, sh-SNHG15#3 plasmid was selected to transfect THP-1 cells. We transfected sh-NC, sh-SNHG15, pcDNA-NC, and pcDNA-SNHG15 into M-CSF and RANKL-induced THP-1 cells, respectively (Figure 2(b)). It was confirmed by CCK-8, TRAP staining, RITC-phalloidin staining, Transwell, and Western blot experiment that knockdown of SNHG15 inhibited the proliferation (Figure 2(c)), generation of osteoclasts (Figure 2(d)), the number of F-actin rings and multinucleate cells (Figure 2(e)), cell migration (Figure 2(f)), and the expressions of osteoclast markers CTSK, MMP-9, NFAT2, and TRAP (Figure 2(g)). The proliferation, generation of osteoclasts, the number of F-actin rings and multinucleate cells, cell migration, and the expressions of osteoclast markers CTSK, MMP-9, NFAT2, and TRAP in the pcDNA-SNHG15group were significantly higher than those in the pcDNA-NC group. To sum up, SNHG15 promoted the differentiation, proliferation, and F-actin production of THP-1 cells induced by RANKL/M-CSF.

### 3.3. SNHG15 Regulated the Expression of NEK2 through Sponging miR-381-3p

**Figure 3:**
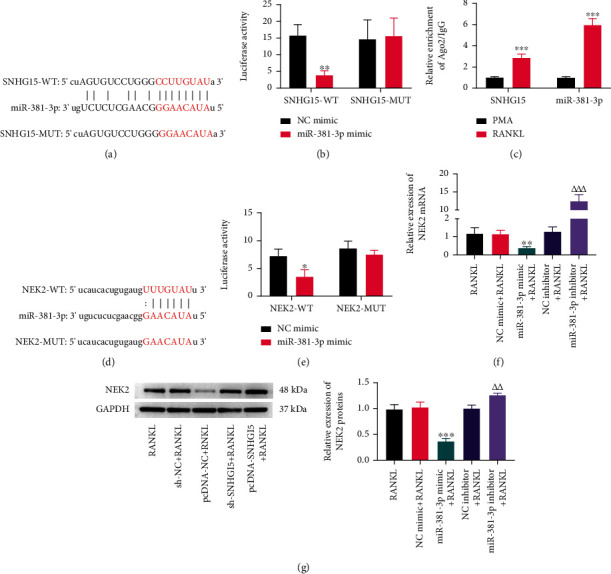
SNHG15 regulates the expression of NEK2 through sponge miR-381-3p. (a) The database predicted the targeted binding sites of SNHG15 and miR-381-3p; (b) the targeting relationship between SNHG15 and miR-381-3p was verified by dual-luciferase reporter gene assay, compared with NC mimic group: ^∗∗^*P* < 0.01; (c) RIP experiments verified the targeting effect of SNHG15 on miR-381-3p, compared with PMA mimic group: ^∗∗∗^*P* < 0.001; (d) the database predicted the targeted binding sites of miR-381-3p and NEK2. (e) The targeting relationship between miR-381-3p and NEK2 was verified by dual-luciferase reporter gene assay, compared with NC mimic group: ^∗^*P* < 0.05; (f) the mRNA expression of NEK2 was detected by RT-qPCR, compared with NC mimic+RANKL group: ^∗∗^*P* < 0.01, compared with NC inhibitor+RANKL group: ^△△△^*P* < 0.001; (g) Western blot was used to detect the proteins expression of NEK2. Compared with NC mimic+RANKL group: ^∗∗∗^*P* < 0.001, compared with NC inhibitor+RANKL group: ^△△^*P* < 0.01.

The starBase database predicted that a targeted binding sequence existed between SNHG15 and the 3′-UTR of miR-381-3p (Figure 3(a)). The dual-luciferase reporter gene assay showed that overexpression of miR-381-3p could significantly inhibit the luciferase activity of wild-type SNHG15, with no statistical difference in the effect on mutant SNHG15 (Figure 3(b)). RIP assay showed that SNHG15 and miR-381-3p were significantly enriched in Ago2 containing miRNA ribonucleoprotein complexes compared with PMA group (Figure 3(c)). It showed that miR-381-3p was the target gene of SNHG15.

Subsequently, we predicted the downstream target gene of miR-381-3p through the starBase database and found that the 3′-UTR of NEK2 had targeted binding sequences with miR-381-3p (Figure 3(d)). Further detection with dual-luciferase reporter gene confirmed that miR-381-3p inhibited luciferase activity of wild-type NEK2, but had no significant effect on mutant NEK2 (Figure 3(e)). RT-qPCR and Western blot assay found that overexpression of miR-381-3p inhibited the mRNA (Figure 3(f)) and protein (Figure 3(g)) expression of NEK2 compared with NC mimic group, and knocking down miR-381-3p promoted the mRNA and protein expression of NEK2. Therefore, miR-381-3p negatively regulated NEK2.

### 3.4. Effects of SNHG15/miR-381-3p/NEK2 Axis on Osteoclast Differentiation, Proliferation, and Movement of Osteoclast Precursors

**Figure 4:**
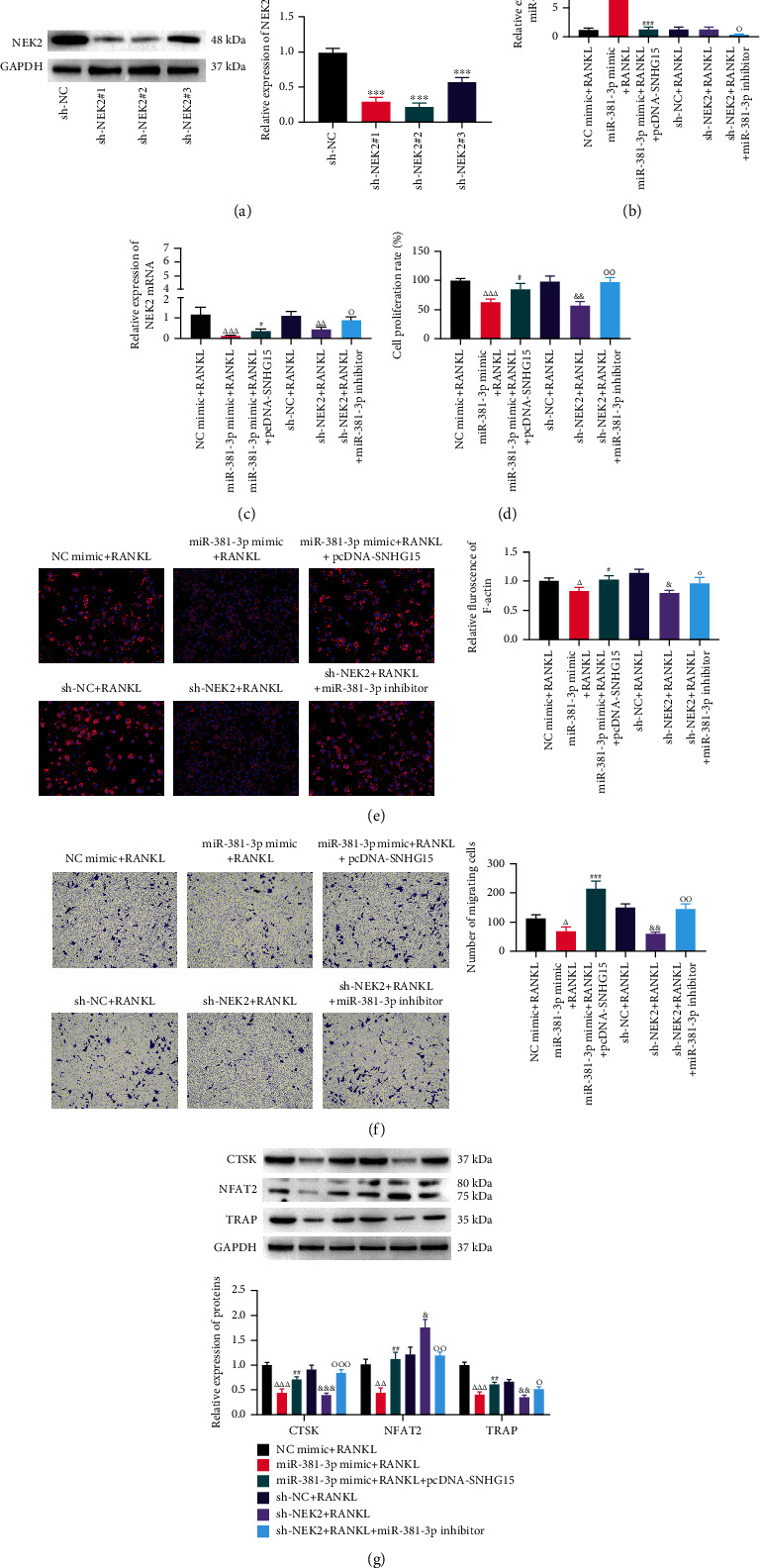
Effects of SNHG15/miR-381-3p/NEK2 axis on osteoclast differentiation, proliferation, and movement of osteoclast precursors. (a) Western blot was used to detect the proteins expression of NEK2; (b) the expression of miR-381-3p was detected by RT-qPCR; (c) The mRNA expression of NEK2 was detected by RT-qPCR; (d) CCK-8 was used to detect cell proliferation rate; (e) rhodamine-labeled phalloidin staining observed the positive rate of F-actin; (f) Transwell detected cell migration; (g) Western blot was used to detect proteins expression of osteoclast markers CTSK, MMP-9, NFAT2, and TRAP; compared with sh-NC group: ^∗∗∗^*P* < 0.001; compared with NC mimic+RANKL group: ^△^*P* < 0.05, ^△△^*P* < 0.01, ^△△△^*P* < 0.001; compared with miR-381-3pmimic + RANKL group: ^#^*P* < 0.05, ^##^*P* < 0.01; compared with sh-NC + RANKL group: ^&^*P* < 0.05, ^&&^*P* < 0.01; compared with sh-NEK2 + RANKL group: ^○^*P* < 0.05, ^○○^*P* < 0.01, and ^○○○^*P* < 0.001.

293T cells were transfected with sh-NEK2#1, sh-NEK2#2, and sh-NEK2#3, respectively. Western blot analysis showed that the expression of NEK2 was significantly inhibited by sh-NEK2 transfection (Figure 4(a)), and sh-NEK2#2 had the best transfection effect. Subsequently, we transfected NC mimic, miR-381-3p mimic, miR-381-3p mimic+pcDNA-SNHG1, sh-NC, sh-NEK2, and sh-NEK2 + miR-381-3p inhibitor into RANKL/M-CSF-induced THP-1 cells, respectively. RT-qPCR detection found that the expression of miR-381-3p was upregulated after transfection with miR-381-3p mimic (Figure 4(b)), the expression of miR-381-3p transfected with miR-381-3p mimic+pcDNA-SNHG15 was significantly lower than miR-381-3p mimic group, and transfection of miR-381-3p inhibitor inhibited the expression of miR-381-3p. Transfection of sh-NEK2 inhibited the expression of NEK2 (Figure 4(c)), and transfection of miR-381-3p mimic+pcDNA-SNHG15 or sh-NEK2 + miR-381-3p inhibitor downregulated the inhibiting effect of transfection of miR-381-3p mimic or sh-NEK2 on NEK2 mRNA expression. CCK-8, RITC-phalloidin staining, Transwell, and Western blot assay revealed that overexpression of miR-381-3p and knockdown of NEK2 significantly inhibited the proliferation (Figure 4(d)), the number of F-actin rings and multinucleate cells (Figure 4(e)), migration (Figure 4(f)), and osteoclast markers CTSK, NFAT2, and TRAP (Figure 4(g)) of THP-1 cells; overexpression of SNHG15 reversed the inhibitory effect of overexpression of miR-381-3p on cell proliferation, the number of F-actin rings, multinucleate cells, migration, and osteoclastic markers. And knockdown of miR-381-3p reversed the inhibitory effect of knockdown of NEK2 on proliferation, the number of F-actin rings, multinucleate cells, migration, and osteoclastic differentiation. In conclusion, overexpression of SNHG15 promoted the expression of NEK2 through sponging miR-381-3p and promoted the proliferation, migration, and differentiation of THP-1 cells induced by M-CSF/RANKL.

## 4. Discussion

Bone is a tissue with active formation and metabolism. The process of bone formation and bone absorption is a continuous process throughout the whole life. Normally, stable metabolism of osteoblasts and osteoclasts ensures the normal structure of bone tissue [20]. But excessive bone resorption can lead to a series of bone-soluble diseases. Therefore, studying the mechanism of osteoclast differentiation will help to understand the pathogenesis of a variety of orthopedic diseases.

Recent studies have shown that abnormal expression of noncoding RNA may involve in the regulation of differentiation of osteoclasts [21]. As one of noncoding RNA, lncRNA plays an important role in various vital activities, including dose compensation effect, epigenetic regulation, and cell differentiation. Chen et al. [22] reported that the expression of lnc BMNCR decreased in RANKL-induced differentiation of osteoclasts *in vitro*, and overexpression of BMNCR significantly inhibited the generation of osteoclasts and bone resorption capacity and reduced the progression of osteoporosis. lnc NRON was a key bone resorption inhibitor, and knocking down NORN in osteoclasts increased bone resorption activity and alleviated osteoporosis [23]; lnc NEAT1 stimulated osteoblast differentiation, and overexpression of NEAT1 promoted the expression of PTK2 through sponging miR-7, accelerated the formation of osteoclasts, and reduced the bone mass of mice [24]. In this study, we found that the expression of SNHG15 in RANKL/M-CSF-induced THP-1 cells was increased, and knocking down SNHG15 inhibited the proliferation and migration of THP-1 cells as well as the expression of osteoclast differentiation markers CTSK, MMP-9, NFAT2, and TRAP. Overexpression of SNHG15 reversed the knock-down effect of SNHG15 on THP-1 cells. This result was consistent with the result reported by Liu and Huang [10] that knocking down SNHG15 inhibited the proliferation of osteoclasts. What is more, SNHG15 has been reported to regulate the proliferation and metastatic of oral squamous cell carcinoma [25], ovarian cancer [26], and bladder cancer [27].

At present, a large number of miRNAs have been confirmed to be involved in the regulation of osteoclast differentiation. For example, the expression of miR-125a-5p was increased in RANKL/M-CSF-induced RAW264.7 cells, and overexpression of miR-125a-5p promoted the differentiation of osteoclasts by targeting TNRSF1B [28]. Osteoblast-derived exosome miR-503-3p inhibited osteoclast differentiation by negatively targeting HPSE [29]. In rheumatoid arthritis, miR-574-5p promoted the maturation of osteoclasts and the development process of rheumatoid arthritis by binding to the activated TLR7/8 signaling pathway [30]. Abnormally expressed miR-381-3p has been reported to play important roles in many diseases, such as tumors [31], spinal cord injury [32], and psoriasis [33]. In this study, we confirmed that miR-381-3p was a target gene of SNHG15 through the starBase database, dual-luciferase reporter gene, and RIP experiment. Overexpression of SNHG15 reversed the inhibitory effect of overexpression of miR-381-3p on the proliferation, migration, and osteoclastic differentiation of THP-1 cells.

Never in mitosis-related kinase 2 (NEK2) is a member of the NEKs family. It is closely related to cell chromosome instability. It is involved in the process of cell centrosome separation, spindle formation, and mitosis. It is highly expressed in multiple myeloma [34], hepatocellular carcinoma [35], and cervical cancer [36]. Hao et al. [34] reported that the expression of NEK2 in multiple myeloma cells increased, positively correlated with the osteolytic lesions in patients with multiple myeloma; the number of osteoclasts in the trabecular region of NEK2 overexpressed mice was increased. NEK2 was overexpressed in cervical cancer tissues and cell lines and was associated with tumor staging and lymph node metastasis in cervical cancer tissues; knocking down NEK2 inhibited the proliferation of cervical cancer and promoted the radiosensitivity of cervical cancer [36]. Hao et al. [34] reported that NEK2 induced osteoclast differentiation and bone destruction via heparinase in multiple myeloma. In this study, we found that NEK2 was the target gene of miR-381-3p, and knockdown of miR-381-3p reversed the inhibiting effect of knockdown of NEK2 on the proliferation, migration, and osteogenic differentiation of THP-1 cells.

To sum up, in this study, we confirmed that the expression of SNHG15 in RANKL/M-CSF-induced osteoclasts was increased, and overexpression of SNHG15 promoted the expression of NEK2, the proliferation, migration, and differentiation of osteoclasts by sponging miR-381-3p.

## Figures and Tables

**Table 1:** Primer sequences.

Target	Sequences (F: forward primer, R: reversed primer; 5′-3′)
SNHG15 [18]	F: GCTGAGGTGACGGTCTCAAA
	R: GCCTCCCAGTTTCATGGACA
GAPDH [18]	F: GAAGAGAGAGACCCTCACGCTG
	R: ACTGTGAGGAGGGGAGATTCAGT
miR-381-3p [19]	F: TCAGACGACAACCGTCTGTG
	R: AAAATTGAGCACCAACGGGC
U6 [19]	F: CTCGCTTCGGCAGCACA
	R: AACGCTTCACGAATTTGCGT

## Data Availability

The datasets supporting the conclusions of this article are included within the article.
